# Isolation and characterization of a CD34^+^ sub-clone in B-cell lymphoma

**DOI:** 10.18632/oncotarget.27415

**Published:** 2020-01-14

**Authors:** Ayad M. Al-Katib, Abdul Shukkur Ebrahim, Mustapha Kandouz, Feras Zaiem, Ali Raufi, Salah Ebrahim, Anwar Mohamed, Nada Emara, Ali M. Gabali

**Affiliations:** ^1^Lymphoma Research Laboratory, Department of Internal Medicine, Wayne State University School of Medicine, Detroit, MI 48201, USA; ^2^Department of Pathology, Wayne State University School of Medicine, Detroit, MI 48201, USA

**Keywords:** CD34, B-cell non-Hodgkin’s lymphoma, minimal residual disease, lymphoma stem cell

## Abstract

Non-Hodgkin’s lymphoma (NHL) is the most common hematological malignancy in the US. Many types remain incurable despite response to initial therapy and achievement of complete remission (CR). Advanced laboratory techniques like multicolor flow cytometry (FCM) and polymerase chain reaction (PCR) have demonstrated persistence of rare malignant cell population post therapy. However, the functional and biological characteristics of this population have not been elucidated.

Established B-lymphoma cell lines (B-NHL) and patient-derived samples (PDS) were analyzed using 8-color FCM. CD34^+^ sub-population was enriched using *in vitro* exposure to 2-chlorodeoxyadenosine (2-CdA) and by CD34 magnetic beads. Genetic analysis of cell fractions was done by karyotyping and array comparative genomic hybridization (aCGH). Sensitivity to chemotherapy was assayed by short-term *in vitro* exposure to chemotherapy. Clonogenicity was determined by soft agar colony formation assay, and proliferation was determined using DNA staining with propidium iodide and FCM.

FCM demonstrated the presence of a minute sub-clone of monotypic B-cells that express CD34 in B-NHL cell lines (3 of 3) and in PDS (8 of 8). This sub-population enriched up to 50 fold *in vitro* by exposure to 2-CdA and up to 80% purity by CD34 magnetic bead column isolation. Except for CD34 expression, this population expressed identical phenotype and genotype to parent cells, but was more proliferative, Hoechst 33342-positive, clonogenic, and resistant to chemotherapy compared with the CD34^-^ population.

The isolated CD34^+^ monotypic B-cells may contribute to resistance of certain NHL to treatment and should be targeted by potential new drugs for NHL.

## INTRODUCTION

Non-Hodgkin’s lymphoma (NHL) is the 7th most common cancer in the United States. More than 74,000 cases were diagnosed in the US in 2018 and almost 20,000 were expected to succumb to the disease [1]. NHL is comprised of a growing list of more than 100 entities based on pathologic, clinical and genetic features [2]. The natural history of NHL is quite variable with some types being slow growing ‘indolent’ (~40%) while others are aggressive or very aggressive [3, 4]. Ironically, the slow growing NHLs remain incurable whereas the aggressive types are curable although 20–40% of those will relapse and may become incurable [5]. All types of NHL are usually responsive to initial treatment and often achieve complete remission (CR), which is a prerequisite to cure. CR is defined as disappearance of lymphoma-related lesions as detected by clinical examination or state-of-the art imaging techniques like PET/CT [6, 7]. Despite achievement of CR, all indolent NHLs and some aggressive ones relapse.

Recurrence of disease is believed to be due to persistence of a relatively small number of lymphoma cells that are below detection level of clinically employed response-evaluation methods. This notion is supported by observations that newer, more sensitive techniques like multicolor flow cytometry (FCM) or polymerase chain reaction (PCR) are able to detect such residual cell population (referred to as minimal residual disease [MRD]) in some NHL patients who are otherwise in CR post completion of therapy [8, 9]. MRD cells carry the same phenotypic and genetic markers as the original lymphoma as detected by FCM and PCR. What makes these cells resist therapy and survive, and how they are different from the rest of lymphoma cells is largely unknown. A study of preclinical mouse model mimicking MRD cells and relapse in acute lymphoblastic leukemia (ALL) patients suggested that drug resistance and dormancy might be linked and represent an acquired stem-like phenotype [10]. Yet, answering such questions may yield insight into developing more effective therapy for NHL. However, the study of MRD in lymphoma has been hampered by cost and lack of MRD monitoring in most prospective clinical trials of NHL [11]. It is encouraging that some of the ongoing clinical trials like the FOLL12 (EudraCT #2012-003170-60) and MIRO trial, EudraCT #2012-001676) have incorporated MRD evaluation.

In this study, we identified a minor cell population within clonal B-cells of lymphoma that expresses CD34 with unique features, including resistance to chemotherapy that distinguishes them from the rest of lymphoma cells. This report describes the isolation process and characterization of this sub-clone.

## RESULTS

### Isolation of CD34^+^ cells

Flow cytometric analysis of all 3 B-lymphoma cell lines revealed the presence of a minor CD34^+^ population. Proportion of these cells was constant during the test period of 72 hours and was <1% (Table 1, Control columns). However this population was enriched several folds after treating cell cultures with 2CdA and progressively increased during the 72 hours of test period. Enrichment was highest in the follicular lymphoma cell line, WSU-FSCCL reaching more than 50 folds at 72 hours compared with control (13.2% vs 0.26%, Table 1), followed by WSU-WM, 10 fold, (2.36% vs 0.23%) and least in the aggressive diffuse large B-cell lymphoma cell line, WSU-DLCL2 (1.25% vs 0.71%).

**Table 1:** Expression of CD34 in human lymphoma cell lines

Percent CD34^+^ Cells						
	**WSU-WM**		**WSU-FSCCL**		**WSU-DLCL2**	
	**Control**	**2-CdA**	**Control**	**2-CdA**	**Control**	**2-CdA**
**24 h**	0.29	0.55	0.21	0.55	0.69	0.86
**48 h**	0.14	1.01	0.2	1.43	0.45	0.51
**72 h**	0.23	2.36	0.26	13.2	0.71	1.25

WSU-WM, WSU-FSCCL, WSU-DLCL2 are human lymphoma cell lines; 24 h, 48 h, 72 h indicate time in culture.

**Figure 1:**
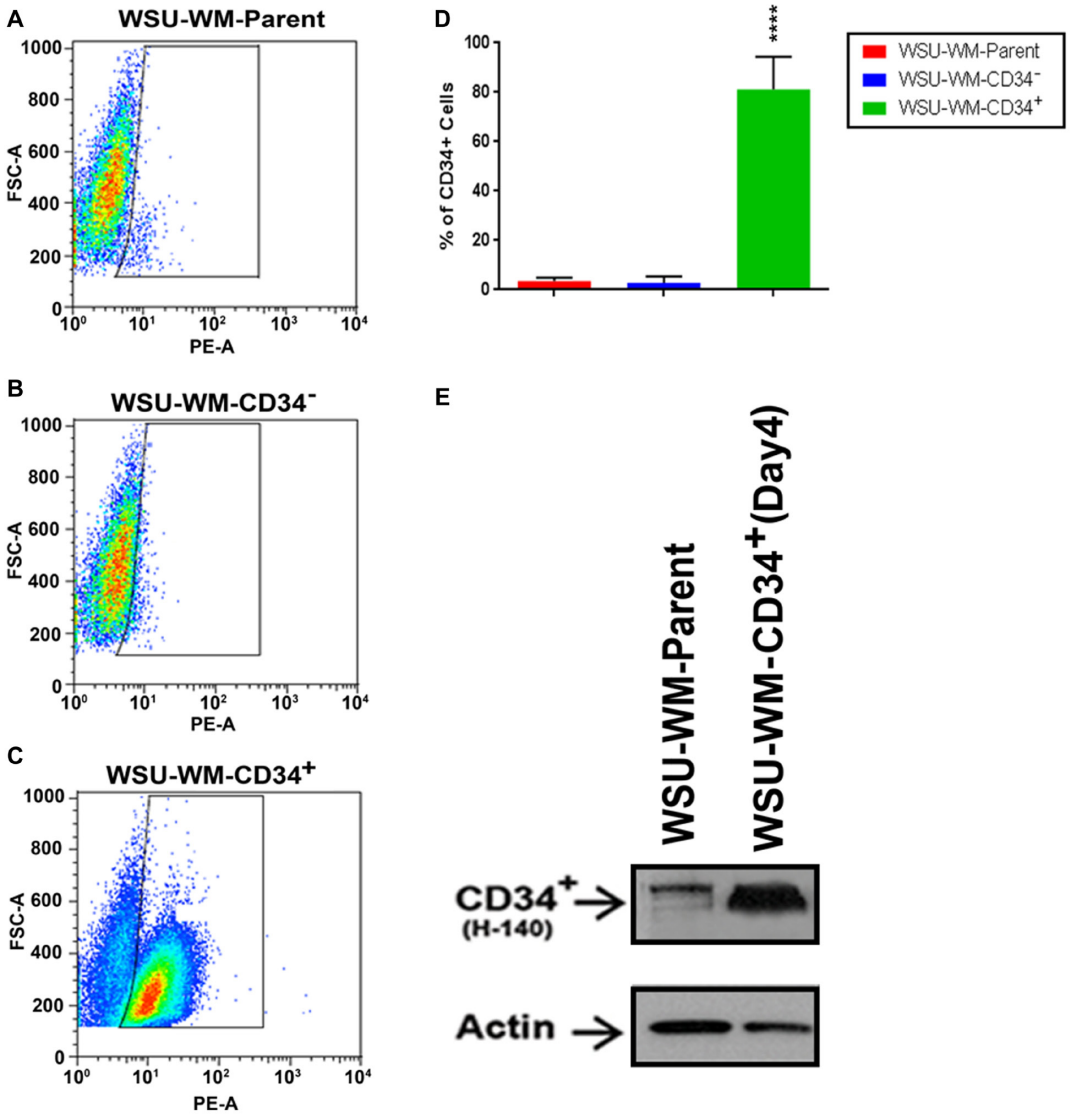
FACS analysis of WSU-WM using CD34 magnetic beads isolation. CD34^+^ subset cells were isolated from human WSU-WM cell line using CD34 Microbeads. Cells were stained with phycoerythrin (PE)-conjugated anti-CD34 antibody (PE-A). (**A–C**) Events are displayed as PE-A vs FSC-A to select singlets. A minor population of CD34^+^ cells was seen in the WSU-WM parent and WSU-WM-CD34^-^ cells (A, B). The CD34^+^ subset population was enriched in the WSU-WM-CD34^+^ cells after bead separation (C). (**D**) Enrichment in WSU-WM-CD34^+^ cells was around (80%). All values represent mean ± SE of five independent experiments. ^****^ *P* < 0.0001 by ANOVA for D. (**E**) Representative Western blots demonstrating CD34^+^ protein expression was increased in WSU-WM-CD34^+^ cell lysates compared with WSU-WM parent cells; an H-140 antibody clone was used to detect CD34; β-actin was used as a loading control.

To enrich the CD34^+^ population further, we applied a CD34 Microbead positive selection technique to 2CdA-treated WSU-WM cell line. As shown in Figure 1, a CD34^+^ cell fraction was enriched up to 80% purity using this method (Figure 1D). Western blots confirmed the higher expression of CD34 protein in the enriched population. These results confirmed the presence of CD34^+^ subpopulation within monotypic B-lymphoma cells and allowed the generation of large number of CD34^+^ cells for further analysis.

### Characterization of CD34^+^ cells

#### Phenotyping

**Figure 2:**
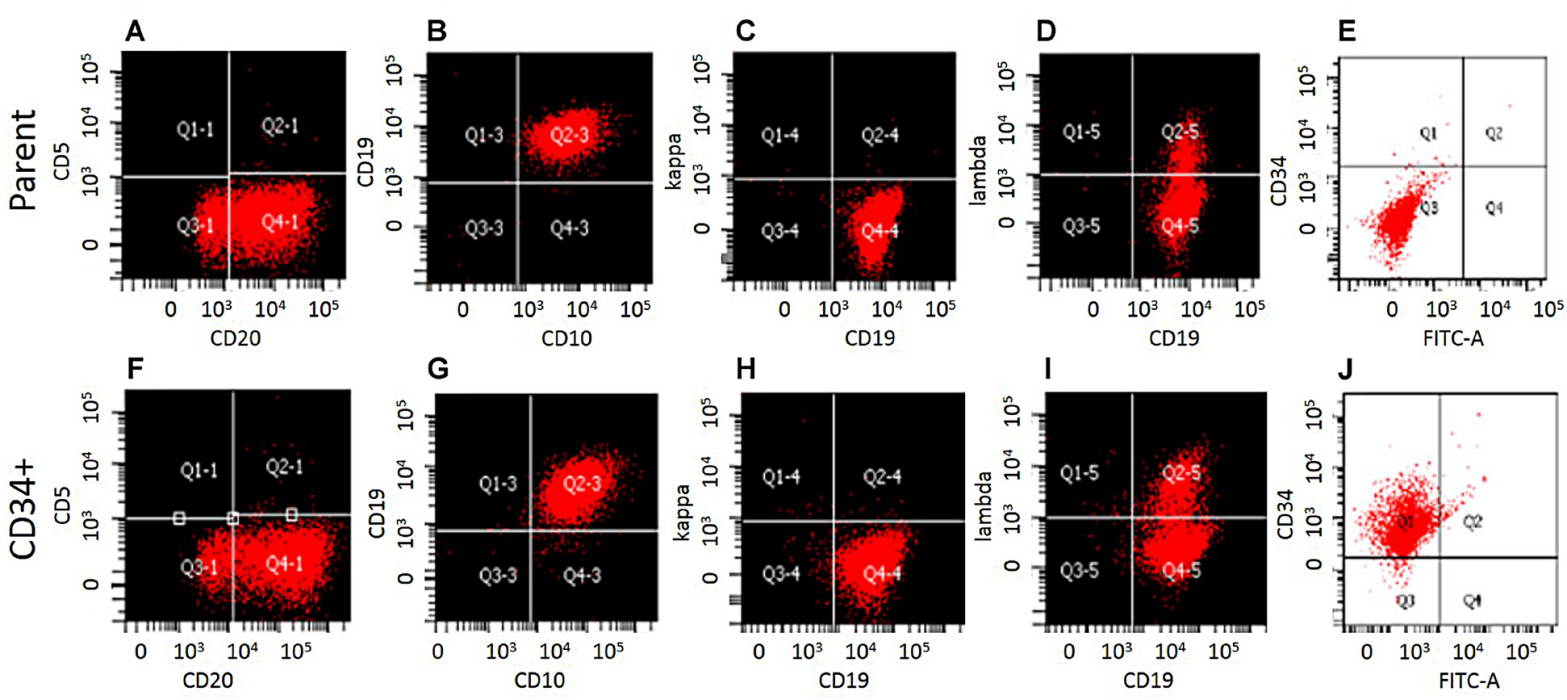
Phenotypic characterization of WSU-WM-CD34^+^ subset cells. Eight color multi parameter flow cytometric analysis of the surface antigen profiles of B-cell markers. (**A–E**), WSU-WM-Parent cells: CD20, CD10, CD19, and Lambda light chain were positive. (**F–J**): CD34 Magnetic bead-isolated cells were positive for CD20, CD10, CD19, Lambda and CD34.

We compared the phenotype of CD34 Microbead-isolated fraction from WSU-WM with parent cells. Except for CD34 expression, the Mirobead-isolated cells exhibited identical phenotype to parent cells as demonstrated by 8-color flow cytometric analysis (Figure 2). Both fractions were clonal B-cells positive for CD10, CD19, CD20 and lambda light chain. This study shows that a subset of mature clonal B-cells can express CD34.

#### Karyotyping and comparative genomic hybridization (CGH) analysis

CD34^+^ cells isolated from WSU-WM also exhibited identical karyotype, SNP, and CGH profile to parent WSU-WM cells (Supplementary Figure 1). By karyotype, WSU-WM-CD34^+^ cells contained 46 chromosomes and exhibited 2p-, t (8;14)(q24; q32), and t (2;17)(q24; q21) translocations as clonal abnormalities (Supplementary Figure 1B). These results were the same as those of parent cells (Supplementary Figure 1A) and as reported in the original characterization of this cell line [12]. Targeted genome SNP profile of WSU-WM-CD34^+^ cells (Supplementary Figure 1C) showed identical pattern of absence of heterozygosity (AOH) as parent cells (Figure 1D). Similarly, whole genome copy number variant (CNV) showed fairly conserved profile of CD34^+^ and parent cells (Supplementary Figure 1E, 1F). Collectively, the findings are indicative of same genetic composition of both cell populations.

#### Hoechst 33342-stained side population (SP) analysis

**Figure 3:**
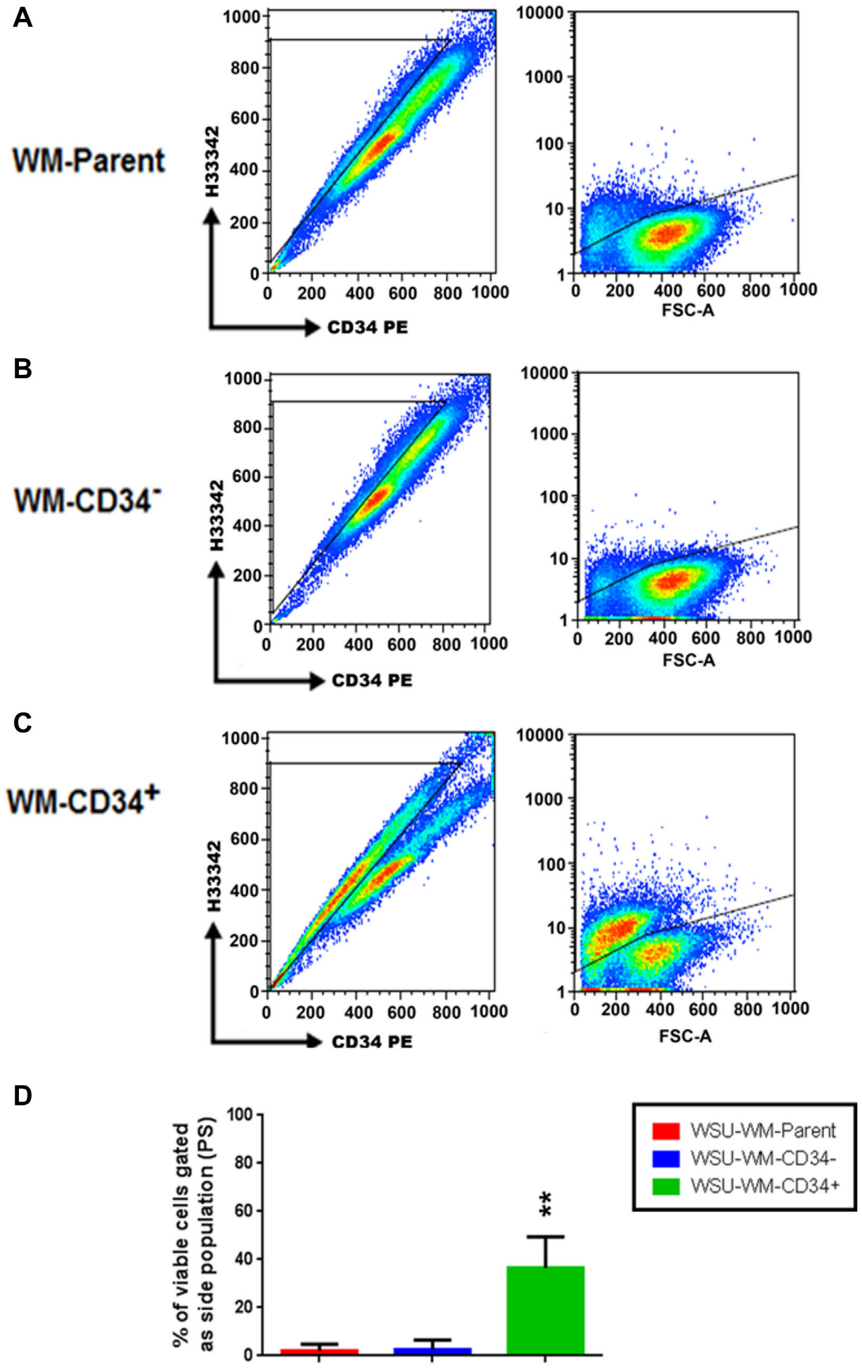
Detection of a side population (SP) in WSU-WM. FACS analysis after Hoechst33342 loading reveals that a few of the SP cells were observed in the parent and CD34^-^ cells (**A**, **B**), but this population was enriched in the WSU-WM-CD34^+^ cells (**C**). The percentage of SP cells in WSU-WM-CD34^+^ was around 40% (**D**). Analysis of representative results from three sets of independent experiments is shown. ^**^ *P* < 0.001 by ANOVA.

FACS analysis of different WSU-WM cell fractions after staining with Hoechst 33342 revealed that only few cells in parent and CD34^-^ fractions were positive (Figure 3A, 3B). In contrast, SP was enriched in the CD34^+^ fraction (Figure 3C). The average number of SP cells in 3 independent experiments was ~40% in the CD34^+^ fraction of WSU-WM (Figure 3D).

#### Growth pattern and clonogenicity of WSU-WM CD34^+^ cells

**Figure 4:**
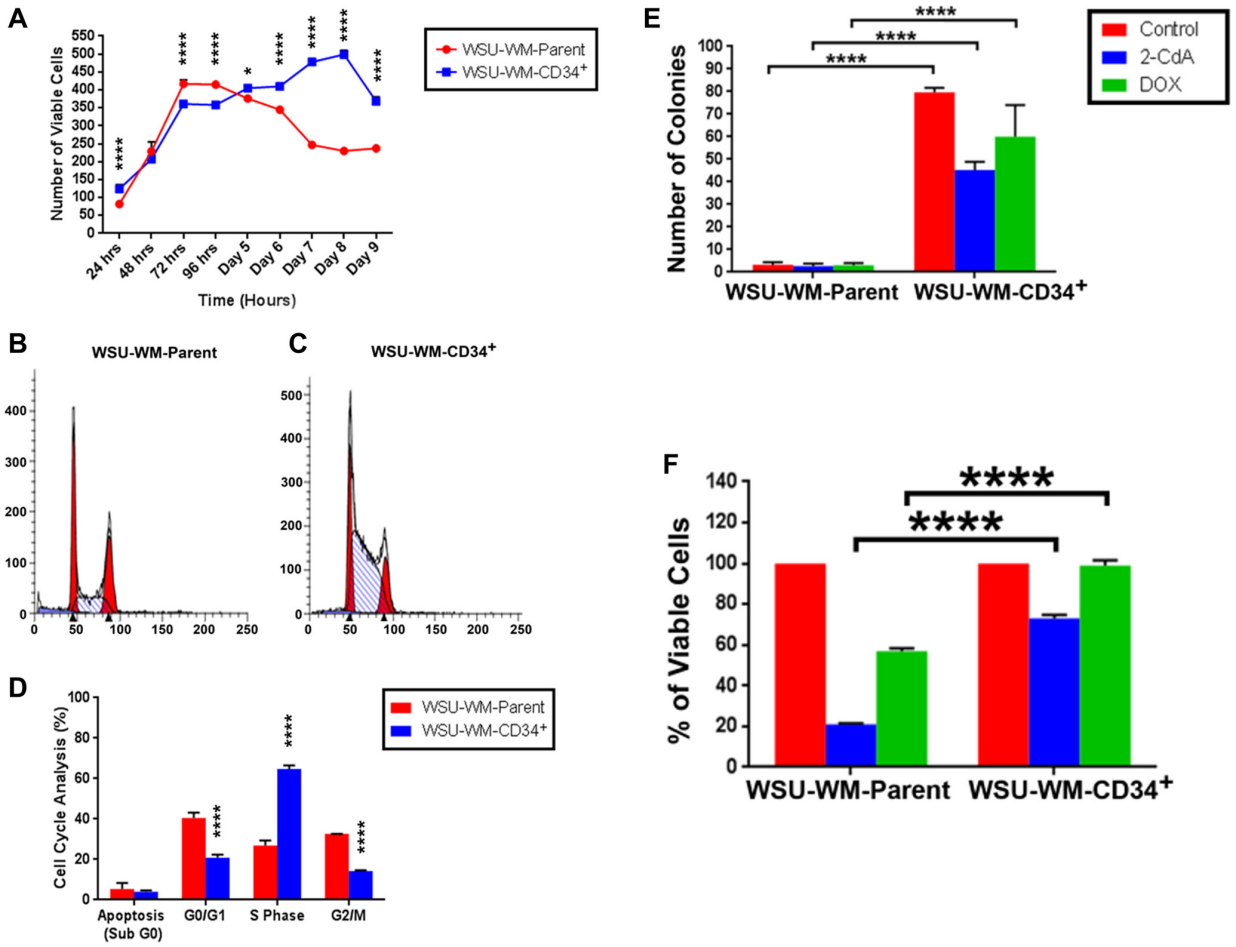
Growth pattern, clonogenicity and chemotherapy resistance of WSU-WM cells. (**A**) Cell viability was measured using 0.4% trypan blue exclusion assay. (**B** and **C**) Flow cytometry data of propidium iodide (PI) staining performed on day 4 of culture in StemPro media. (**D**) Cell cycle distribution of WSU-WM parent and CD34^+^ cells (mean ± SE of triplicate experiments). (**E**) Clonogenicity of parent and CD34^+^ cell fractions in soft agar in presence of cytotoxic chemotherapy agents. Cells were treated with 2-CdA at 50 nM, or Doxorubicin (DOX) at 1.5 pM for 48 hours in liquid culture then suspended in RPMI-1640 medium containing 0.35% noble agar and plated in 6-well plates at a density of 5 × 10^3^/well. (**F**) Cell viability in presence of cytotoxic chemotherapy agents after 5 days in liquid culture. Drug concentrations were same as in (E).

Using StemPro media, CD34^+^ WSU-WM fractions showed more sustained viability in culture over 9 day period compared with parent cells (Figure 4A). Moreover, CD34^+^ cells exhibited different growth pattern compared with parent cells. The growth curves separated after the 4th day where the CD34^+^ cells demonstrated continued increase in cell number whereas parent cells were decreasing in number. Cell cycle analysis of the two cell subsets supported the growth pattern in cell culture. CD34^+^ cells exhibited higher percentage of cells in S phase compared with parent cells (Figure 4B–4D). Moreover, CD34^+^ cells were more clonogenic even in presence of chemotherapy agents, 2-CdA and doxorubicin compared with parent cells (Figure 4E) and demonstrated resistance to cell kill by these agents in liquid culture (Figure 4F). Expression of CD34+ cells decreased with time and was ~2% on day 9 of culture in the StemPro media.

### Detection of CD34^+^ cells in patient-derived samples

Eight patient-derived samples were included in this study. The cases were selected retrospectively from the clinical laboratory data based on the following criteria: 1) A final diagnosis of mature B-cell malignancy, 2) Involvement of blood by the malignant process, 3) Inclusion of CD34 antibody in the staining panel. Patient data is summarized in Table 2 and details of 8-color flow cytometry data of the first 6 cases is shown in Supplementary Figure 2. Two of the cases (#1 and #6) had a clear diagnosis of chronic lymphocytic leukemia/small lymphocytic lymphoma (CLL/SLL). Case #1 had stage 0 disease by Rai classification and case #6 had stage I. One case (#2) had Richter’s transformation and high WBC count (163.2 × 10^3^/µL) and one (#8) had a diffuse large B cell lymphoma. Both of these cases had advanced stage disease (stage IV) by virtue of involving the blood. The remaining four cases were diagnosed as B-cell lymphoproliferative disorder (B-cell LPD) without further characterization.

**Table 2:** CD34 expression in patient-derived blood samples

Patient# Age/Sex	Diagnosis	Positive Immunophenotype	Karyotype/FISH	WBC/ALC	CD34%
#1 37/M	CLL/SLL	CD5, CD11c, CD19, CD20, CD23, CD25, CD45, lambda	Deleted 13q14	24.1 × 10^3^/ul 15 × 10^3^/ul	0.01%
#2 73/M	Richter’s syndrome	CD5, CD11c, CD19, CD20, CD23, CD25, CD45, kappa	N/A	163.2 × 10^3^/ul 66.1 × 10^3^/ul	5.35%
#3 72/M	B-Cell LPD	CD19, CD20, CD23, CD45, FMC7, kappa	Deleted 13q14 Deleted TP53/17p13	12 × 10^3^/ul 4.3 × 10^3^/ul	0.19%
#4 74/M	B-Cell LPD	CD5, CD19, CD20, CD23, CD45, FMC7, kappa	Deleted TP53/17p13	N/A	1.72%
#5 74/F	B-Cell LPD	CD5, CD11c, CD19, CD20, CD23, CD45, kappa	N/A	N/A	0.35%
#6 65/M	CLL/SLL	CD5, CD11c, CD19, CD20, CD23, CD38, CD45, kappa	Trisomy 12	20.9 × 10^3^/ul 19 × 10^3^/ul	0.01%
#7 77/M	B-Cell LPD	CD5, CD11c, CD19, CD22, CD38, FMC7, HLA-DR, kappa	Deleted 13q14 Deleted TP53/17p13	21.1 × 10^3^/ul 4.1 × 10^3^/ul	0.02%
#8 67/F	DLBCL	CD5, CD10, CD11c, CD19, CD20, CD38, CD79a, FMC7, HLA-DR, kappa	N/A	47.5 × 10^3^/ul 10.4 × 10^3^/ul	0.25%

Abbreviations: B-Cell LPD, B-cell lymphoproliferative disorder; CLL/SLL, chronic lymphocytic leukemia/small lymphocytic lymphoma; DLBCL, diffuse large B cell lymphoma; F, female; M, male; N/A, not available; WBC/ALC, white blood cell count/absolute lymphocyte count.

CD34 expression was determined specifically on the clonal B-cells (Supplementary Figure 2) and ranged between 0.01% and 5.35%. Given the small number of cases, no conclusion can be drawn regarding factors that influence CD34 expression level. However, the highest expression (5.35%) was seen in the case of Richter’s syndrome. Overall, the patient-derived data lend support to, and was consistent with the cell line data.

## DISCUSSION

In this report, we provide evidence for the existence of a minor subpopulation within mature malignant B-cells that expresses different features from the rest of cells. This population appears to have the same genetic composition, as determined by karyotype and CGH, and to express the same phenotype as the rest of cells. However, they exhibit distinct characteristics giving them potential important role in resistance to chemotherapy and incurability of disease. The distinguishing phenotypic marker of this subpopulation is expression of CD34. This CD34^+^ clonal B-cell subpopulation was identified in established B-cell lymphoma cell lines (3 of 3) (Table 1) and in all patient-derived samples (8 of 8) (Table 2).

Although CD34 is considered a marker of normal hematopoietic stem cells and for some acute myeloid leukemia, its expression is not limited to such cells. CD34 expression on non-myeloid, malignant hematopoietic cells was reported before. For example, CD34 was shown to be expressed in 24/45 of adult acute lymphoblastic leukemia (ALL) and was associated with Pgp-MDR1 phenotype and worse outcome compared with 21/45 CD34 negative cases [19]. Only 11/24 (45%) of CD34 positive cases achieved CR after induction chemotherapy compared with 20/21 (95%) of CD34 negative cases. Survival was significantly shorter (6.6m vs 13.5m). A recent study highlights the importance of using multiple markers to detect MRD in B lymphoblastic leukemia [20]. Two case control studies of B-ALL patients and control participations at diagnosis and after 2–3 weeks of induction chemotherapy suggested that CD34/CD123 [21] and CD10/CD19/CD34 with CD45 or CD97 [22] are more specific for MRD detection in B-ALL. In another study, clonal CD34^+^CD19^+^ progenitors were detected in bone marrow of BCL2-IgH-positive follicular lymphoma patients [23]. Our study shows that such cells (CD34+CD19+) are not limited to follicular lymphoma since we were able to detect them in diffuse large B-cell lymphoma (WSU-DLCL2), Waldenström’s macroglobulinemia (WSU-WM), chronic lymphocytic leukemia/small lymphocytic lymphoma (CLL/SLL) and other B-cell lymphoproliferative disorders (fresh patient-derived samples).

CD34 protein is a member of a family of single-pass transmembrane sialomucin proteins that is expressed on early hematopoietic and vascular-associated tissue [24]. Its function overlaps with other CD34-related proteins, podocalyxin and endoglycan [25, 26]. These related proteins are also expressed on early hematopoietic progenitor cells. However, how these related molecules contribute to progenitor and stem cell function and behavior needs further investigation [27].

Clinically, CD34 is not considered part of the diagnostic antibody panel for lymphoma. Therefore, studies addressing minimal residual disease (MRD) in lymphoma do not determine if there is differential expression of this marker in MRD cell population [28, 29]. Another technique for detection of MRD in lymphoma is based on PCR for known chromosomal translocations like the t (11;14), t (14;18) or immunoglobulin heavy chain genes (IgH) [30, 31]. Newer molecular technologies such as droplet digital PCR and next generation sequencing have also been described for monitoring MRD in B cells lymphoproliferative disease [32]. PCR technique, like the flow cytometry will not detect the CD34+ population within MRD since CD34+ cells have the same genetic composition as parent cells, at least by karyotype and CGH analyses (Supplementary Figure 1). We hypothesize that CD34+ cells are epigenetically modified in order to respond and adapt to external signals. Comprehensive gene sequencing efforts in follicular lymphoma, for example, lends support to our hypothesis. A study by Okosun et al. [33] demonstrated recurrent mutations in linker histones, and chromatin regulators (*CREBBP, EZH2*, and *MLL2*) as early driver genes occurring in a common progenitor clone (CPC). The authors concluded that the mutational landscape of follicular lymphoma is predominantly epigenetic. However, it is possible that CD34+ cells do have genetic aberrations not detectable by karyotype or CGH given the limitations of these techniques [34].

In order to obtain large number of CD34+ cells and characterize this cell population further, we sought to enrich the CD34+ population in our cell lines using 2 strategies, 2-CdA and CD34 magnetic beads separation. Our initial enrichment step using *in vitro* exposure to the chemotherapy agent 2-CdA simulates clinical MRD setting. Such strategy showed up to 50 fold enrichment of the CD34+ population (Table 1) suggesting that MRD cells in clinical studies are likely to preferentially express CD34. We believe that adding CD34 to MRD antibody panel in clinical studies of B-cell lymphoma will be useful in capturing this population. It should be emphasized, however, that our study is performed in established cell lines and in patient-derived samples of limited number. Therefore, larger prospective study of patient-derived samples is needed to establish the presence of CD34+ cell fraction in MRD samples at diagnosis and after successful treatment.

The mechanism of 2-CdA-induced CD34+ cell enhancement is not clear. However, we postulate that 2-CdA induced preferential killing of CD34 negative cells leading to a relative increase in CD34+ cells. This postulation is based on the hypothesis that CD34+ are more resistant to chemotherapy as part of their ‘stem cell’ characteristics. Our experimental data showed good inverse correlation between cytotoxicity and CD34+ cells after exposure to chemotherapy *in vitro*. In one experiment, exposure of WSU-WM cells to 2-CdA for 72 hours resulted in 5.7 fold reduction in cell viability and a 5.9 fold increase in the percentage of CD34+ cells (data not shown).

Although the CD34+ population has identical phenotype, except for CD34 expression, and genetic composition to parent cells, they exhibited important differences in some of their characteristics and biological behavior. Some of these features are similar to those described for tumor initiating cells. For example, 43% of the CD34+ cells in our WSU-WM cells were also Hoechst 33314+ (Figure 3). This sub-population, also known as side population (SP), was described before in different human solid tumors [35] and in leukemia [36]. It was shown to initiate tumors in mantle cell lymphoma [37]. SP cells also demonstrated resistance to chemotherapy in Hodgkin lymphoma but, interestingly, were susceptible to elimination by novel therapies like immunotherapy [38] and NF-κB inhibition [39]. Consistent with these observations, we have reported earlier that a novel experimental therapeutic agent, SAR3419 (a CD19 antibody-drug conjugate) was far more effective against our WSU-FSCCL and WSU-DLCL2 xenograft models than standard chemotherapy regimen, CHOP or rituximab [16]. It is possible, although not proven, that such enhanced activity is due to targeting the CD34+ population –demonstrated in both of these models- which are also CD19+.

Other features of the CD34+ sub-population isolated in our studies are enhanced survival, clonogenicity and proliferation and resistance to chemotherapy *in vitro* (Figure 4). Although it is commonly believed that stem cells are usually quiescent, there is evidence that some tumor initiating cells are proliferative [40]. Enhanced cell survival in culture media that supports stem cells (StemPro^®^) (Figure 4A) and in presence of chemotherapy agents (Figure 4F) as well as clonogenecity in presence of chemotherapy agents (Figure 4E) are all indications of a unique cell population that is likely to be clinically significant.

Collectively, our results indicate that the sub-population of CD34+ clonotypic B-cells that we isolated is a candidate lymphoma stem cell (LSC). The concept of LSC is not new [41, 42]. However, definitive evidence for its existence has not been provided. Part of the difficulty is the definition of LSC, or cancer stem cell in general. It is also not clear if LSC is a fixed cell type or represent multiple compartments. Our finding that only a subset of CD34+ cells is SP+ suggests heterogeneity within the CD34+ population. Such model allows LSC to adapt to external environment by switching between proliferative and survival mode in order to evade therapy. It is imperative, therefore that future drug development in lymphoma focus on this cell population as target. Ability to isolate and enrich such cells from established cell lines as we did provides a mechanism for testing experimental therapeutic agents against these cells.

## MATERIALS AND METHODS

### Cell culture

Three human B- lymphoma cell lines were used in this study, WSU-WM [12], WSU-FSCCL [13], and WSU-DLCL2 [14]. The WSU-WM cell line is an IgM-secreting lymphoplasmacytic lymphoma established from a patient with Waldenström’s macroglobulinemia (WM). The WSU-FSCCL cell line was established from a patient with follicular small cleaved cell lymphoma (FSCCL) which is equivalent to follicular lymphoma, grade 1 by WHO classification [15]. The WSU-DLCL2 represents an aggressive diffuse large cell lymphoma (DLCL). All cell lines were established by the Lymphoma Research Laboratory at Wayne State University (WSU), Epstein-Barr virus (EBV)-negative, well characterized and authenticated. Cells are maintained in RPMI-1640 medium supplemented with 10% fetal bovine serum (FBS), 1% L-glutamine and antibiotics as previously described [16]. CD34^+^ cells were also cultured in StemPro^®^-34 SFM medium. This is a serum-free medium (SFM) containing StemPro^®^- 34 nutrient supplement (Gibco by Life Technologies) specifically formulated to support the development of human hematopoietic cells in culture.

### Patient-derived samples

Blood specimens analyzed in the clinical laboratory for suspected diagnosis of hematopoietic malignancy and worked up for both leukemia and lymphoma diagnosis were reviewed. Cases that had final diagnosis of a B-cell malignant process, and had CD34 marker included in the antibody panel were included in this study. Final diagnosis was confirmed by expert Hematopathologist, (AG). An 8-color flow cytometry using a FACS Canto II from Becton Dickinson (San Jose, CA, USA) was conducted on these blood samples. Both leukemia and lymphoma staining protocols (Supplementary Table 1) as described below were applied since the diagnosis was not clear at the time of testing.

### Surface phenotyping

Three and 8 color flow cytometry analysis was conducted on cell lines and patient-derived samples to confirm B-cell clonality, and to identify and characterize CD34^+^ cells. Cultured cells were collected from the media by centrifugation in 15 ml tube for 15 minutes at 300 g at room temperature. Cell pellets were washed using phosphate-buffered saline (PBS) and then suspended in PBS with 30% heat inactivated Adult Bovine Serum (ABS) (Atlanta Biologicals Inc.; Flowery Branch, GA, USA) to block non-specific binding. After performing cell counts and viability, as described below, cells were centrifuged and the pellets were incubated with CD19-FITC, CD10-PE, and CD34-PC5, or Isotype controls (Beckman Coulter Inc.; Brea, CA) using manufacturer recommended volumes and incubation conditions. Cells were washed briefly in PBS and the pellets were suspended and fixed in 0.4 ml of 0.4% formaldehyde-PBS solution (Polysciences Inc.; Warrington, PA, USA) prior to flow cytometry event acquisition using Coulter flow cytometer (Beckman Coulter Inc.; Brea, CA, USA). The fluorescence intensity of CD19, CD10 and CD34 antigens of 10,000 cells were analyzed after gating on intact cells using forward and light-scatter patterns. Boolean gating strategies were employed to gate the CD34 positive and CD34 negative fractions, which were then analyzed for CD19 and CD10 expression.

For patient-derived specimens, an 8-color flow cytometry using a FACS Canto II from Becton and Dickinson (BD; San Jose, CA, USA) was used. A lymphoma and acute leukemia staining protocols were applied to peripheral blood samples using 8-color antibody panels as shown in Supplementary Table 1. In contrast to the leukemia staining protocol, the lymphoma protocol is preceded by two steps that include a prewash to remove excess specimen immunoglobulin that may interfere with the monoclonal antibodies and incubation with normal rabbit serum (NRS) to neutralize non-specific binding sites on cell surface. Briefly, two washes are done by adding 15 ml of wash media (0.9% NaCl, 0.1% Na-azide and 10% Heat inactivated fetal calf serum, HI-FCS) to 300 μl of whole blood, centrifuged at 300 x *g*, at room temperature, for 3 minutes and decant thoroughly. After the second decant, 1 ml of NRS is added to the cell pellet, mixed gently, and incubated at 37°C for 30 minutes. After incubation, cells are washed and resuspended in up to 500 μl of RPMI with 5% HI-FCS. After these two steps, the lymphoma and leukemia staining protocols are similar. Briefly, 100 μl of whole blood obtained from individuals with non-Hodgkin’s B-cell lymphoma patients were incubated with the appropriate antibodies for 10 minutes at 4°C in the dark. Cells were further processed using a standard lysed whole blood procedure by adding 2 ml FACS lysing solution (BD, San Jose, CA, USA), vortexed gently, and then incubated in the dark for 10 min at room temperature. The cells were centrifuged at 300 × *g* for 5 minutes at room temperature, washed twice in wash media and the pellets resuspended in 0.5 ml of wash media and the analyzed within 4 hours. In general, a minimum of 10,000 events/cells was acquired.

### 2-Chlorodeoxyadenosine (2-CdA) treatment

6 × 10^6^ cells of WSU-WM, WSU-FSCCL, and WSU-DLCL2 in 5 mL culture media were exposed to 100 nM concentration of 2-CdA or PBS (control) for up to 72 hours. Cell count and viability was determined every 24 hours as described below. Simultaneously, 3 color flow cytometry was conducted at each time point as described above to enumerate CD34^+^ cells.

### CD34 magnetic bead isolation

10^8^ cells of WSU-WM cells in T-75 tissue culture flasks were exposed to 2-CdA at 100 nM for 48 hours and cell count and viability are determined. Cells were then processed for the isolation of CD34^+^ cells using CD34 MicroBead Kit UltrePure (Miltenyi Biotech, Auburn, CA) following manufacturer protocol. Briefly, cells were incubated with 100 µL of CD34 MicroBeads for 30 minutes at 4°C, washed, resuspended in 500 µL buffer and applied onto MS column placed in magnetic field of MACS Separator. CD34^+^ cells are pulled towards the internal surface of the column through the iron magnetic beads attached to the CD34 antibody. The column is then rinsed 3 times with 500 µL of buffer to collect flowing through CD34-unlabeled cells. Column is then removed from the separator and CD34-labeled cells are pushed out using a plunger, resuspended in 1mL of buffer. Collected cell fractions are then stained with CD34PE (#130-081-002, Miltenyi Biotech) and analyzed by flow cytometry.

### Soft agar colony formation assay

The colony formation assay was conducted to assess clonogenic potential of different cell fractions as described previously [12]. In brief, Control (WSU-WM parent cells), and CD34^+^ WSU-WM cells were treated with the cytotoxic drugs Doxorubicin (1.5 pM) and 2CdA (50 nM) for 48 hrs. Single cell suspensions in complete RPMI medium containing 0.35% noble agar (Difco, Maryland) were plated at a density of 5 × 10^3^ cells in six-well plates coated previously with 0.6% base agar and maintained at 37°C in a humidified atmosphere containing 5% CO2 for 14 days. Colonies >0.2 mm in diameter were counted in eight random fields and normalized to control cells.

### Cell cycle analysis

Flow cytometric analysis of propidium iodide (PI)-stained cells was conducted to assess the cell cycle under different experimental conditions. WSU-WM-parent, -CD34^+^, and -CD34^-^ cells were collected and centrifuged twice in cold PBS. Cells were then fixed in 5 ml of 70% ethanol and stored at 4°C overnight [17]. Cells were centrifuged and resuspended in 1 ml of staining buffer containing 50 ug/ml propidium iodide, 100 ug/ml of RNase A, and 0.1% of Triton X-100. DNA content was analyzed on a Coulter EPICS 753 flow cytometer and different stages of the cell cycle were determined using a ModFit 5.2 computer program. The flow cytometry work was done at the Microscopy, Imaging and Cytometry Resources Core at Wayne State University.

### Side population (SP) analysis

The fluorescent vital dye Hoechst 33342 binds DNA in living cells and was shown to stain hematopoietic cells that have stem cell characteristics [18]. We therefore sought to evaluate our CD34+ cell fraction for staining characteristics with this dye. WSU-WM cell suspensions were labeled with Hoechst 33342 dye (Invitrogen by ThermoFisher Scientific). Briefly, cells were re-suspended at 1 × 10^6^/mL in pre-warmed RPMI with 10% FBS medium. Hoechst 33342 dye was added at a final concentration of 5 μg/mL and the cells were incubated at 37°C for 90 min with intermittent shaking. At the end of the incubation, cells were washed with ice-cold PBS and analyzed on a Coulter EPICS 753 flow cytometer.

### Cell count and viability

Cells were seeded at a density of 0.2 × 10^6^ viable cells/mL per well in a 24-well plate (Costar, Cambridge, MA, USA) for variable periods of time. The number of viable cells was determined by trypan blue exclusion (Sigma Chemical Co. St. Louis, MO, USA) at 24 hour intervals [17].

### Immunoblotting

Cells were harvested, washed in PBS and lysed in M-PER lysis buffer containing a protease and phosphatase inhibitor cocktail (Thermo Scientific, Rockford, IL, USA) [17]. Equal amounts of protein lysates were subjected to SDS-PAGE followed by blotting with the indicated antibodies and detection by Western Super Signal West Pico Chemiluminescent substrate reagents (Thermo Scientific, Rockford, IL, USA). Images were quantified using ImageJ densitometry software (Version 1.45, US National Institutes of Health) and normalized to the actin signal used as internal control. Data are presented as relative band signal intensity compared to control.

### Cytogenetics

WSU-WM and WSU-WM-CD34^+^ cells in log phase of growth were exposed to colcemid (0.05 pg/mL) for 1 hour. Cells were then harvested and treated with 0.075 mol/L potassium chloride for I5 minutes before they were fixed with 3:1 methanol:acetic acid. Slides were prepared by dropping cell suspension onto ethanol-cleaned glass microscope slides. Q and G-banding methods were applied in the staining of chromosomes. From each culture, 20 to 30 cells were analyzed microscopically and karyotypes prepared.

### Comparative genomic hybridization (Array-CGH)

Chromosomal microarray (CMA) using the oligonucleotide-single nucleotide polymorphism (Oligo-SNP), whole genome Agilent 180K GGXChip+SNP (Agilent Technologies, Inc) was performed. The approximate distance between probes is 80kb with a distance of 20kb in targeted regions. Thresholds for the detection of deletions and duplications are 6 probes with an average value of .25 and .20 respectively. The test is useful for the detection of DNA copy number losses or gains associated with chromosomal imbalances at multiple targeted sites throughout the genome. In addition this test may detect copy neutral aberrations such as uniparental disomy (UPD) and absence of heterozygosity (AOH). This array does not detect balanced reciprocal translocations, Robertsonian translocations, inversions, balanced insertions, point mutations, or imbalances of regions not represented on the microarray.

### Data analysis and statistics

Statistics were conducted using GraphPad Prism 5.0 for Windows (GraphPad Software) and tests were done with one-way ANOVA (^*^ *P* < 0.05, ^**^ *P* < 0.01, ^***^ *P* < 0.001). *P* values less than 0.05 were considered to be statistically significant. Results are displayed as averages with error bars indicating standard deviations (SD). ImageJ densitometry software (Version 1.45, US National Institutes of Health) was used for quantification of Western blot bands. Selected bands were quantified based on their relative integrated intensities, calculated as the product of the selected pixel area and the mean gray value for those pixels normalized to the internal control.

## SUPPLEMENTARY MATERIALS


