# Supplementary material for: Isolation and characterization of a CD34+ sub-clone in B-cell lymphoma
Source: Oncotarget. 2020 Jan 14;11(2):148–60. doi: 10.18632/oncotarget.27415 (PMC6968783; doi:10.18632/oncotarget.27415)
Supplement: Supplementary file 1 [file oncotarget-11-148-s001.pdf]

Isolation and characterization of a CD34^{+} sub-clone in B-cell lymphoma

SUPPLEMENTARY MATERIALS

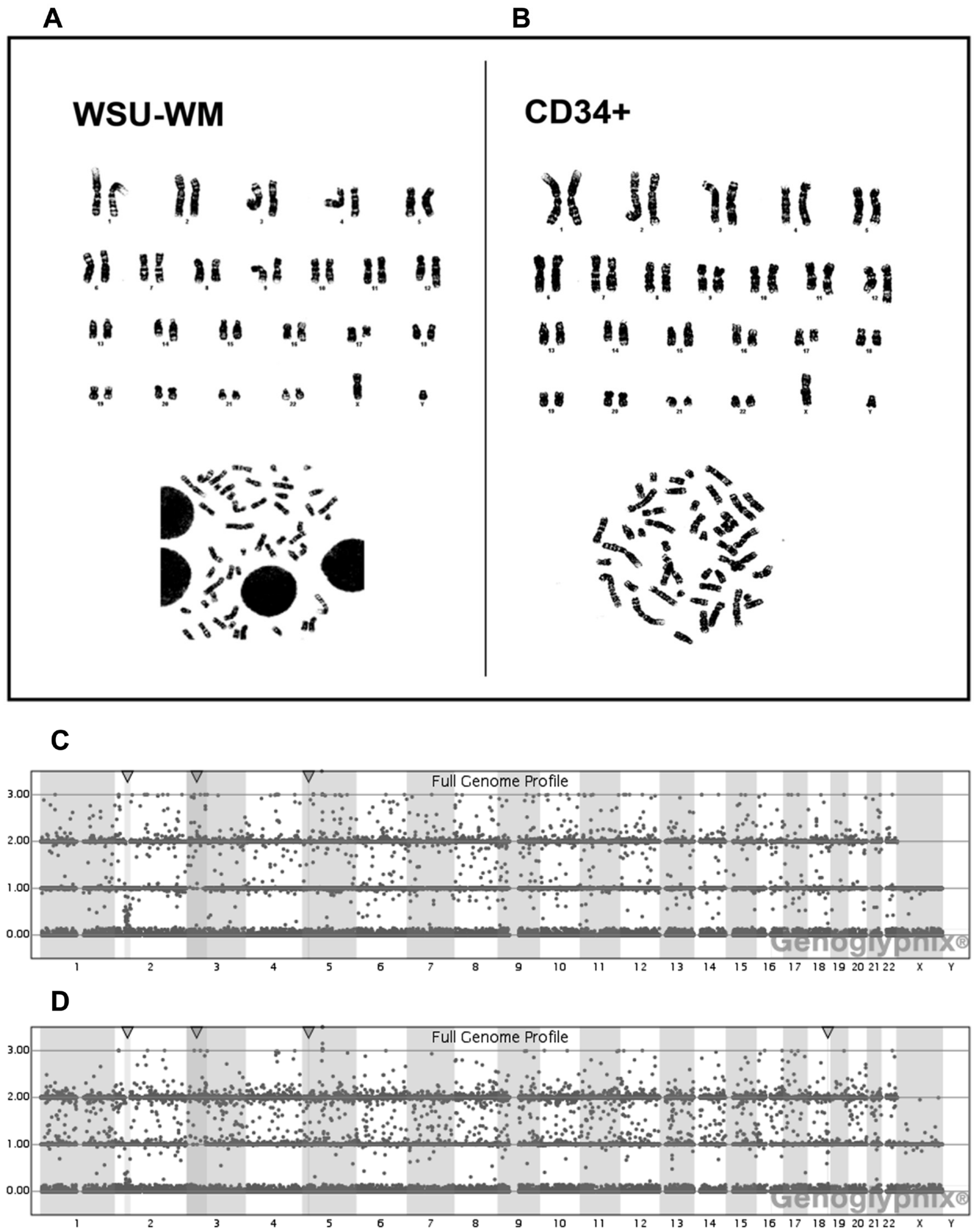

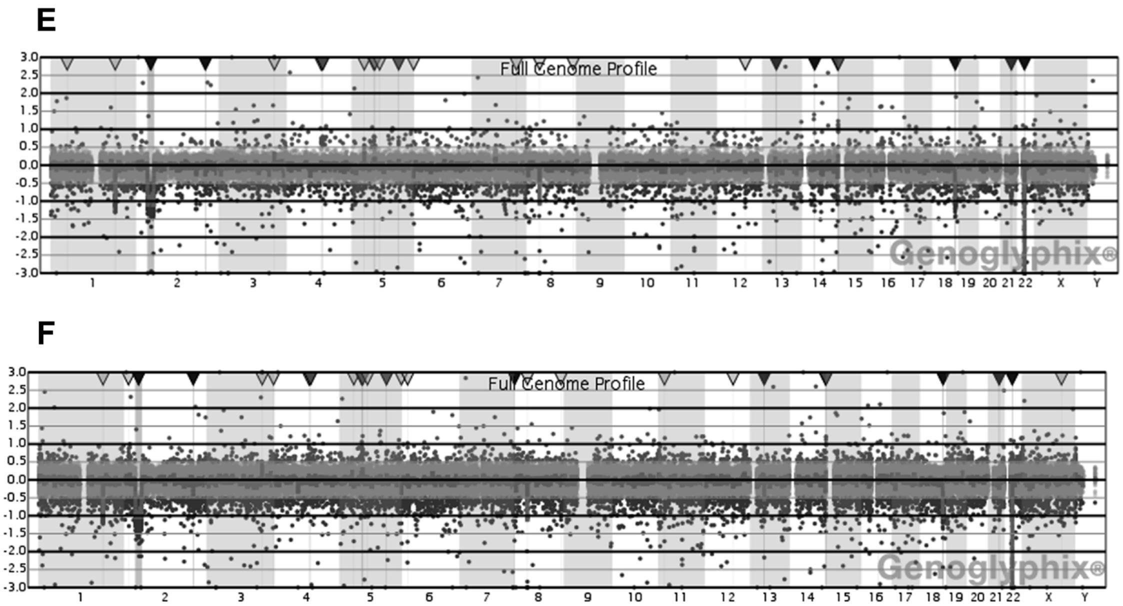

Supplementary Figure 1: Genetic characterization of CD34+ WSU-WM cells. (**A**, **B**): G-banded karyotype of WSU-WM parent cells (A) and WSU-WM CD34+ cells showing the same abnormalities in both cell subtype (See text for description of abnormalities). (**C**, **D**): A Targeted-genome SNP profile of CD34+ WSU-WM cells (C) and parent cells (D) depicting an identical pattern of absence of heterozygosity (AOH) indicative of same genetic composition, where the x-axis coordinates represent chromosomes 1-22, X, Y (p arm to the left, q arm on the right) and the marked, shaded areas represent AOH. (**E**, **F**): A whole-genome copy number variant (CNV) profile of CD34+ WSU-WM cells (D), and parent cells (F) showing fairly conserved CNV profile indicative of same genetic composition, with the x-axis representing chromosomes 1-22, X, Y (p arm to the left, q arm on the right). Deletions and duplications are marked and show a negative or positive log value shift respectively.

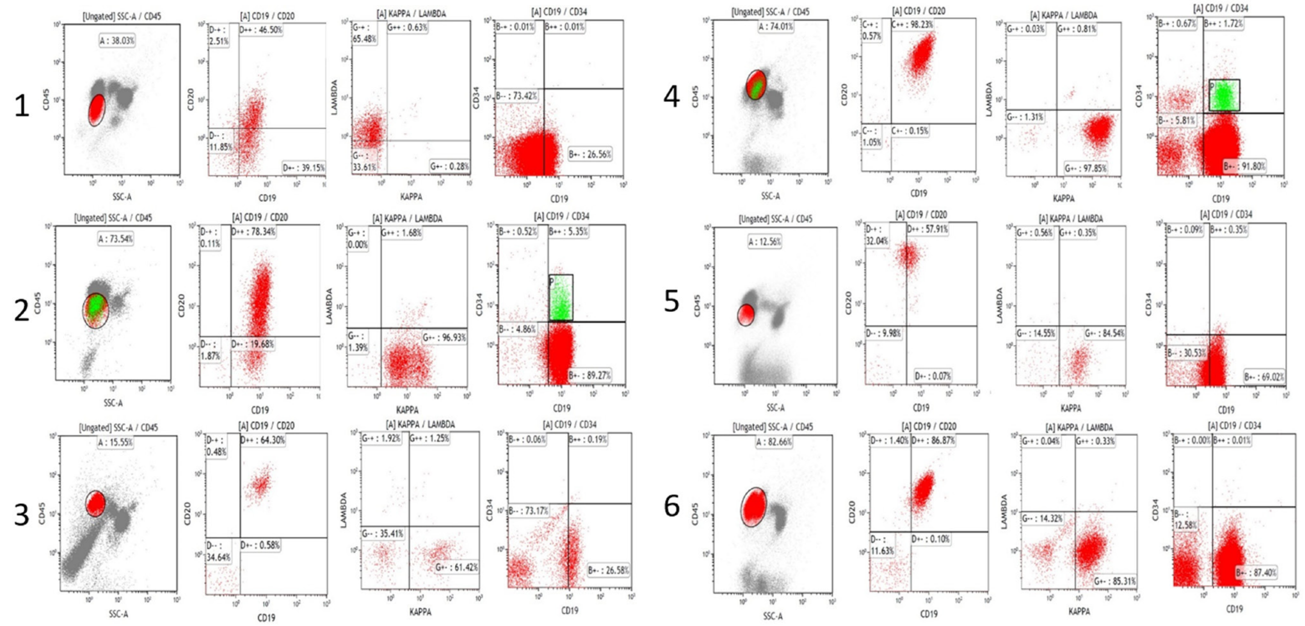

Supplementary Figure 2: Flow cytometry data of patient-derived samples. The data is summarized in Table 2. Numbers 1–6 are patient numbers as appear in Table 2.

Supplementary Table 1: Lymphoma (upper) and leukemia (lower) antibody panels used for 8-color flow cytometry

	**FITC**	**PE**	**PCPC5.5**	**PC7**	**APC**	**APCH7**	**V450**	**V500C**
TUBE 1	LAMBDA	KAPPA	CD5	CD10	CD2	CD19	CD20	CD45
TUBE 2	CD8	CD2	CD5	CD4	CD25	CD3	CD7	CD45
TUBE 3	CD103	CD23	CD5	CD38	CD11C	CD19	FMC7	CD45
TUBE 4	CD103	CD22	CD5	CD25	CD11C	CD19	CD7	CD45

	FITC	PE	PCPC5.5	PC7	APC	APCH7	V450	V500C
TUBE 1	CD14	CD13	CD33	CD117	CD34	CD19	CD7	CD45
TUBE 2	CD22	CD11C	CD5	CD10	CD34	CD19	CD38	CD45
TUBE 3	CD36	CD64	HLADR	CD56	CD34	CD4	CD16	CD45
